# LINE Retrotransposon RNA Is an Essential Structural and Functional Epigenetic Component of a Core Neocentromeric Chromatin

**DOI:** 10.1371/journal.pgen.1000354

**Published:** 2009-01-30

**Authors:** Anderly C. Chueh, Emma L. Northrop, Kate H. Brettingham-Moore, K. H. Andy Choo, Lee H. Wong

**Affiliations:** Chromosome and Chromatin Research Laboratory, Murdoch Children's Research Institute, Melbourne University Department of Paediatrics, Royal Children's Hospital, Parkville, Victoria, Australia; Medical Research Council Human Genetics Unit, United Kingdom

## Abstract

We have previously identified and characterized the phenomenon of ectopic human centromeres, known as neocentromeres. Human neocentromeres form epigenetically at euchromatic chromosomal sites and are structurally and functionally similar to normal human centromeres. Recent studies have indicated that neocentromere formation provides a major mechanism for centromere repositioning, karyotype evolution, and speciation. Using a marker chromosome mardel(10) containing a neocentromere formed at the normal chromosomal 10q25 region, we have previously mapped a 330-kb CENP-A–binding domain and described an increased prevalence of L1 retrotransposons in the underlying DNA sequences of the CENP-A–binding clusters. Here, we investigated the potential role of the L1 retrotransposons in the regulation of neocentromere activity. Determination of the transcriptional activity of a panel of full-length L1s (FL-L1s) across a 6-Mb region spanning the 10q25 neocentromere chromatin identified one of the FL-L1 retrotransposons, designated FL-L1b and residing centrally within the CENP-A–binding clusters, to be transcriptionally active. We demonstrated the direct incorporation of the FL-L1b RNA transcripts into the CENP-A–associated chromatin. RNAi-mediated knockdown of the FL-L1b RNA transcripts led to a reduction in CENP-A binding and an impaired mitotic function of the 10q25 neocentromere. These results indicate that LINE retrotransposon RNA is a previously undescribed essential structural and functional component of the neocentromeric chromatin and that retrotransposable elements may serve as a critical epigenetic determinant in the chromatin remodelling events leading to neocentromere formation.

## Author Summary

The centromere is an essential chromosomal structure for the correct segregation of chromosomes during cell division. Normal human centromeres comprise a 171-bp α-satellite DNA arranged into tandem and higher-order arrays. Neocentromeres are fully functional centromeres that form epigenetically on noncentromeric regions of the chromosomes, with recent evidence indicating an important role they play in centromere repositioning, karyotype evolution, and speciation. Neocentromeres contain fully definable DNA sequences and provide a tractable system for the molecular analysis of the centromere chromatin. Here, the authors investigate the role of epigenetic determinants in the regulation of neocentromere structure and function. They identify that a retrotransposable DNA element found within the neocentromere domain is actively transcribed and that the transcribed RNA is essential for the structural and functional integrity of the neocentromere. This study defines a previously undescribed epigenetic determinant that regulates the neocentromeric chromatin and provides insight into the mechanism of neocentromere formation and centromere repositioning.

## Introduction

Despite the fact that the functional role of the centromere in mitotic and meiotic cell divisions is evolutionarily conserved, the underlying DNA sequences of the centromeres are highly variable across the phylogeny and show no obvious conservation [1],[2]. Thus, a conundrum remains as to whether there are any specific sequence requirements for the different types of, primarily tandemly repeated, DNA in providing the template for centromere formation. In recent years, accumulating evidence has pointed to epigenetic factors including DNA methylation and histone modifications as having important roles in the establishment of centromeric chromatin [3],[4]. In addition, the discovery of fully functional human neocentromeres that arise ectopically from non-tandemly repetitive chromosomal sites further supports the fundamental roles of epigenetic phenomena in the regulation of centromere activity [5]. This class of variant centromeres not only represents an apparently sequence-independent epigenetic model for centromerization but also serves as an excellent tool for the detailed mapping of centromeric chromatin domains – an undertaking that has previously been hampered by the repetitive nature of the mammalian centromeric DNA [6].

The core neocentromeric chromatin is fundamentally defined by the presence of specialized centromere-specific histone H3 variant CENP-A nucleosomes; however, the exact molecular mechanisms involved in the formation of a neocentromere have yet to be defined [7],[8],[9],[10]. To date, approaching one hundred cases of neocentromere emergence have been reported on all the human chromosomes except for chromosomes 7, 19, and 22 [6]. Interestingly, some genomic regions, such as the terminal chromosomal segments of 3q, 8p, 13q, and 15q, are more prevalent in neocentromere cases, with these ‘hotspots’ collectively accounting for approximately half of all the cases reported [5],[11]. Although the ectopic emergence of neocentromeres *in hitherto* non-centromeric genomic sites suggests the involvement of epigenetic mechanisms of formation, it remains possible that the underlying genomic DNA sequences exert a specific role in the establishment and/or maintenance of the functional integrity of the neocentromeric chromatin. For example, such a possibility is suggested by the universal observation of an elevated AT content, an increase in the density of LINEs (Long Interspersed Nuclear Elements), and a decrease in the density of SINEs (Short Interspersed Nuclear Elements) for the six different neocentromeric domains that have been mapped to date [7],[8],[9],[10].

The first human neocentromere was identified at position 10q25 on the derivative marker chromosome mardel(10) following a *de novo* interstitial pericentric deletion that has removed the presiding centromere of a normal chromosome 10 [12]. Despite the lack of detectable α-satellite DNA, the 10q25 neocentromere was able to form a mitotically stable kinetochore that binds over 40 of the known functionally important centromere-associated proteins tested [13],[14],[15],[16]. Using a combined BAC (Bacterial Artificial chromosome)-array/ChIP (Chromatin Immunoprecipitation) technique, the CENP-A-associated domain was mapped to a 330-kb genomic segment along the 10q25 neocentromeric chromatin [9]. Subsequently, other centromere protein-binding domains such as those of HP1 and CENP-H, and an increased scaffold/matrix attachment region (S/MAR), were mapped, defining an overall neocentromeric chromatin region of approximately 4.0 Mb in size [17].

To further define the finer structural organization of the core neocentromeic chromatin, we have recently performed high-resolution chromatin mapping using PCR fragment-array/ChIP analysis. The CENP-A domain was found to be assembled as multiple clusters (seven in total) along the 10q25 neocentromeric chromatin [18]. Interestingly, *in silico* sequence analysis indicated that these CENP-A-binding clusters contain a 2.5-fold increase in the prevalence of L1 retrotransposon sequences (which belong to the only active subfamily of LINEs) when compared to the surrounding non-CENP-A-binding regions or the genome average [18],[19],[20]. L1 retrotransposon is a major group of interspersed repetitive elements that comprise 17% of the human genome. Although the great majority of L1s are inactive due to 5′ end truncations, active transcription and translation of these retrotransposons has recently been detected in a variety of cell types and implicated to be a potential regulator for cellular processes [19],[20]. However, detailed investigations on the functional role of individual L1 retrotransposon in the human genome have been limited by technical difficulties associated with its repetitive nature. In this study, we present an in-depth bioinformatic analysis and the experimental investigation of the possible functional roles of the L1 retrotransposons in the regulation of neocentromere activity.

## Results

### Enrichment of L1 Retrotransposons at the 10q25 Neocentromeric Chromatin

**Figure 1:**
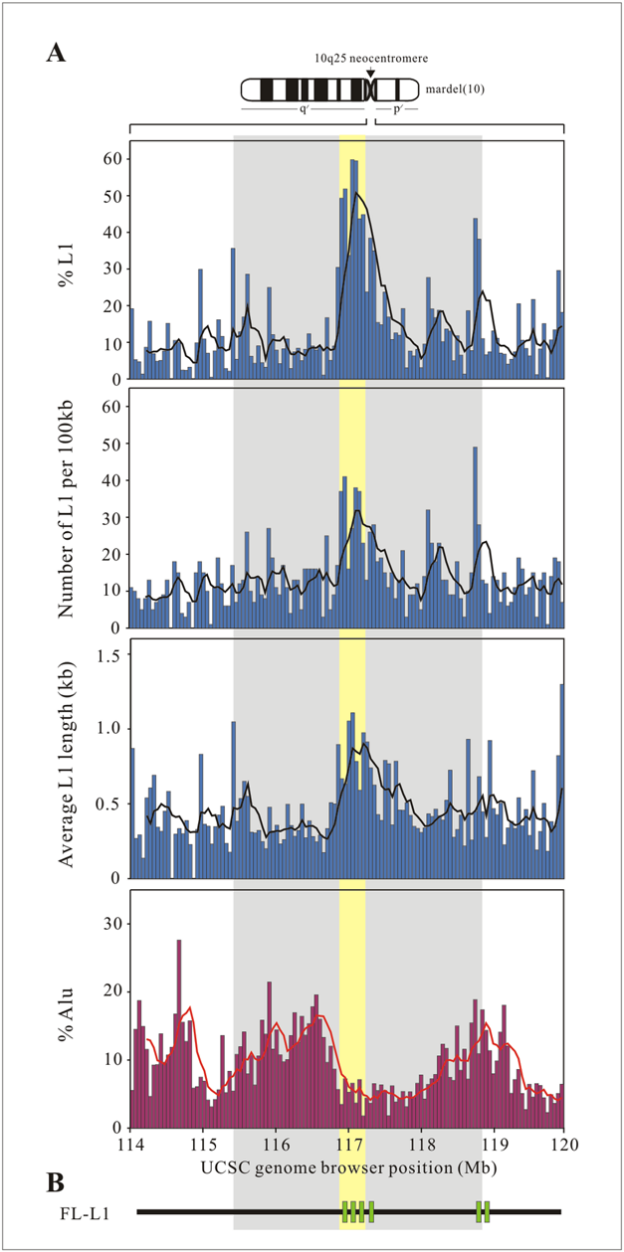
*In silico* analysis of the 10q25 neocentromere DNA. (A) Average abundance and length of L1 (or Alu) sequences along a 6-Mb genomic segment spanning the 10q25 neocentromere, encompassing the 330-kb CENP-A-binding domain (shaded in yellow; see Figure 2B for distribution of the seven CENP-A-binding sub-clusters within this domain) and the 3.5-Mb increased chromosomal scaffold (S/MAR)-attachment domain (shaded in grey) [17] analyzed using a 50-kb window. (B) Distribution of full-length L1s (green bars) as identified using the online L1Base program (http://l1base.molgen.mpg.de/).

Our previous *in silico* analysis of the various types of DNA motifs and sequence properties revealed a significant, 2.5-fold, increase in the prevalence of L1 retrotransposons within the CENP-A-binding domain of the 10q25 neocentromere [18]. Here, we extended the analysis to the investigation of the genomic distribution and sequence characteristics of L1 retrotransposons across a 6-Mb genomic region spanning the 10q25 neocentromere using the RepeatMasker track of the UCSC genome browser. Besides an enrichment of L1 retrotransposons, the CENP-A-binding clusters of the 10q25 neocentromere were also associated with a higher number of intact L1 genomic segments (Figure 1A). These CENP-A-binding clusters contained 56 L1s per 100 kb DNA, whereas the flanking non-CENP-A-binding regions contained only 26 L1s per 100 kb DNA, with an overall 2.1-fold increase in L1 content in the CENP-A-binding regions (Table S1). In addition to the bioinformatics analysis, ChIP/quantitative PCR analysis using a specific antibody against CENP-A also showed a specific enrichment of L1 genomic sequences in the CENP-A-associated chromatin of 10q25 neocentromere ( Figure S1).

**Figure 2:**
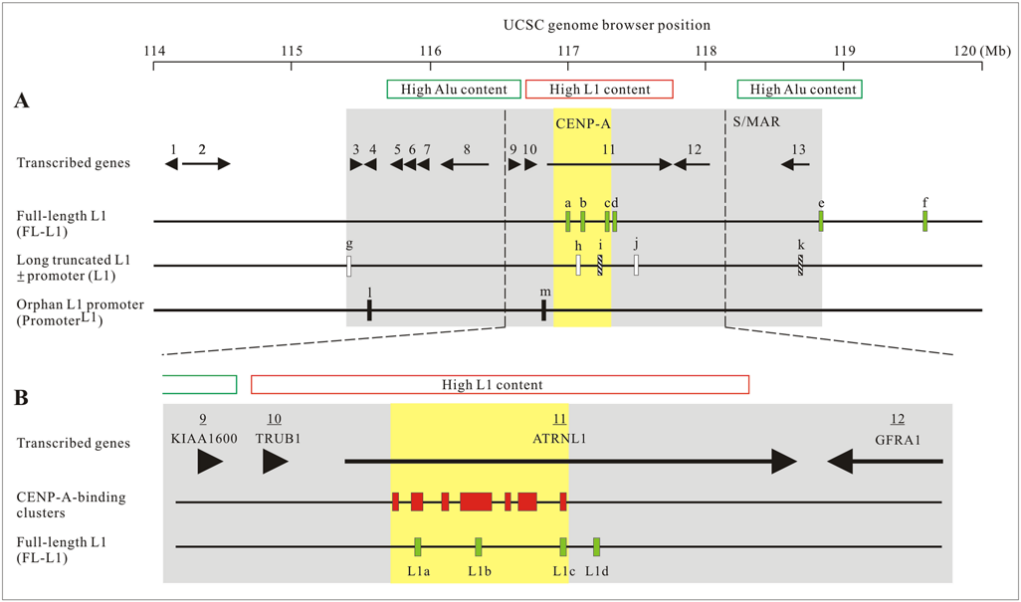
Distribution of FL-L1s, long truncated L1s, and orphan L1 promoters across a 6-Mb genomic region of the 10q25 neocentromere. (A) A total of six FL-L1s (green bars) were found, with a cluster of four of these elements (a–d) localizing within and near the CENP-A-binding region (shaded in yellow) [9]. Open bars denote long truncated L1s; hatched bars denote truncated L1s containing an internal promoter sequence; black bars denote orphan L1 promoter sequences. The positions of 13 transcribed genes (black arrows/arrowheads) are shown [17], of which 11 are found within the 3.5-Mb S/MAR domain (shaded in grey). (B) Close-up view of the CENP-A-binding and immediately surrounding region, showing the distribution of the seven CENP-A-binding clusters in relation to the ATRNL1 gene and FL-L1a–d. Note that FL-L1a–c reside within the CENP-A-binding clusters (red boxes) [18], with the actively transcribed FL-L1b being localized within the largest, central cluster.

Although there was no significant difference in term of the rate of divergence, deletion, and insertion between the L1 retrotransposons within the CENP-A and non-CENP-A-associated regions across the 6-Mb region of the 10q25 neocentromere (Table S1), the average length of the L1 retrotransposons located within the CENP-A-binding regions (average length of 865 bp) was significantly longer (increased by 2 folds) compared with those found within the non-CENP-A-binding regions (average length of 440 bp) (Figure 1A; Table S1). Such a difference was attributed to an increase in the proportion of the primate-specific L1 subfamily, as shown by a higher L1P/L1M ratio (L1P, primate-specific; L1M, mammalian-wide), within the region. Given the L1P subfamily included active full-length L1 (FL-L1) retrotranposons, we next searched for the presence of FL-L1 at this region. Functional annotation of the FL-L1 retrotransposons spanning across the 6-Mb region of the 10q25 neocentromere using the online L1Base program (http://l1base.molgen.mpg.de/) identified six FL-L1s, four of which (L1a–d) residing within or close to the CENP-A-associated clusters, while the remaining two (L1e–f) were located >1.5 Mb away from the CENP-A-associated domain (Figure 1B and Figure 2).

### Active Transcription of a FL-L1 Sequence within the CENP-A-Binding Domain

**Figure 3:**
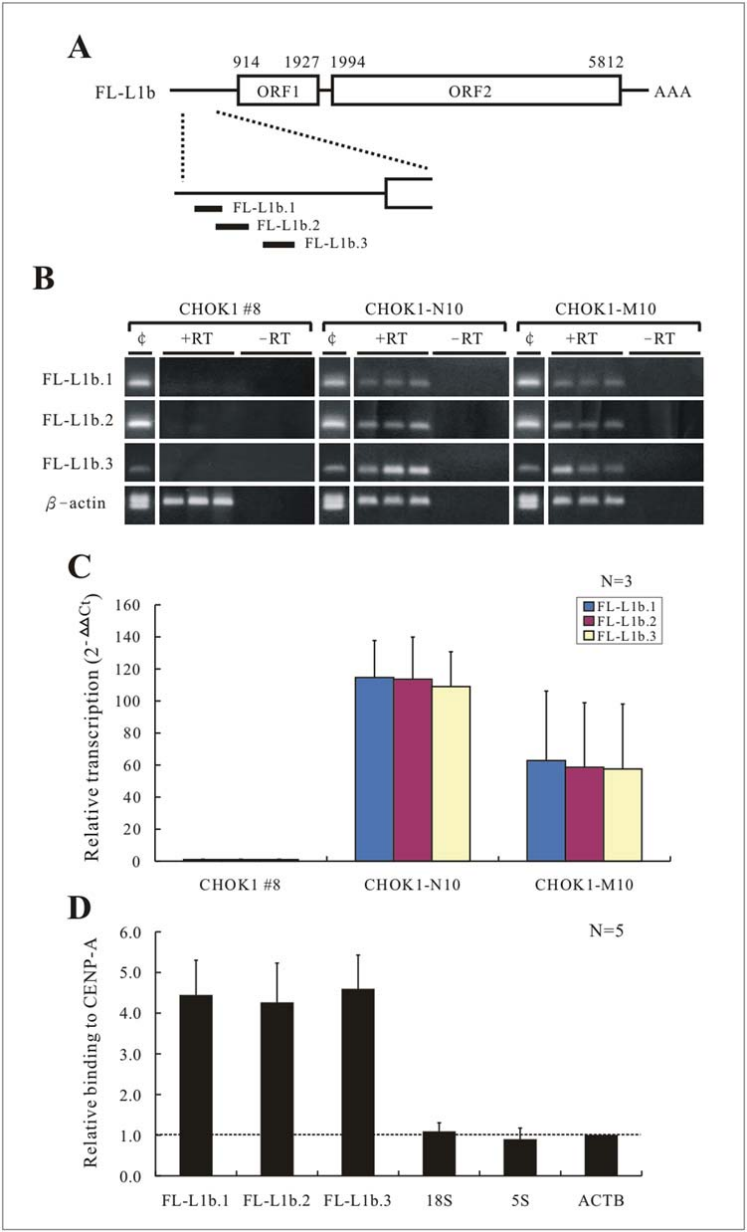
FL-L1b transcription and RNA-ChIP analysis. (A) A schematic diagram showing the FL-L1b target sites for the three independent primer sets (FL-L1b.1, FL-L1b.2, and FL-L1b.3; together spanning a genomic region of 415 bp) used in RT-PCR assays. (B) Positive transcription of FL-L1b was seen with all 3 primer sets in CHOK1-N10 and CHOK1-M10 cell lines, but not in CHOK1 #8 cell line after 40 cycles of RT-PCR amplification. CHOK1 #8, CHOK1-N10 and CHOK1-M10 were hamster-human monochromosomal hybrid lines containing unrelated normal human chromosome 10, progenitor normal human chromosome 10, and mardel(10) chromosome, respectively. The transcription of positive control β-actin gene was detected in all the cell lines. ‘¢’ = genomic DNA control. (C) Quantitative RT-PCR results (mean for n = 3, with standard error of the mean, SEM), indicating no significant difference in FL-L1b transcription in CHOK10-N10 and CHOK1-M10. Relative FL-L1b transcription levels in CHOK1-N10 and CHOK1-M10 were compared to that of CHOK1 #8, which was used as a normalization control. ΔC_T_ = C_T_ [test segment]−C_T_ [β-actin control]. (D) RNA-ChIP-qPCR analysis. ChIP was performed using an anti-CENP-A antibody followed by quantitative RT-PCR analysis. FL-L1b RNA was significantly enriched (*P*<0.05) in the CENP-A-bound fraction in CHOK1-M10 but not in CHOK1-N10. None of the negative controls (18S rDNA, 5S rDNA, and β-actin ACTB RNAs) was enriched in the precipitated fractions. Relative binding values (mean for n = 5, with SEM) on the Y-axis represent the fold-enrichment of FL-L1b RNA CHOK1-M10 compared to that of CHOK1-N10.

Although the functional role of L1s in the regulation of genomic architecture is not well defined, it is of significant interest that L1s can be transcribed into RNA and subsequently translated into proteins for retrotransposition activity [20],[21],[22],[23]. Recent reports indicate that L1 RNAs are actively transcribed in a variety of cell types from full-length L1 elements (∼6 kb in size) that contain an internal promoter, two ORFs, and a poly-A tail at the 3′ UTR [20],[21],[22],[23]. To address if any of the six FL-L1s at the 10q25 neocentormere chromatin were transcriptionally active, RT-PCR primers were designed to specifically target each of the elements (L1a–f) in monochromosomal CHO-human hybrid lines, CHOK1-M10 and CHOK1-N10 (containing the human neocentromeric mardel(10) and the progenitor normal human chromosome 10, respectively) (Figure 3). The specificity of each primer was confirmed by direct sequencing of the PCR products, which established that only the desired target sites were amplified.

No transcripts from FL-L1a, FL-L1c, FL-L1d, FL-L1e and FL-L1f were detected. However, as shown in Figure 3A–C, based on the use of three independent primer sets that targeted to a combined genomic segment of 415 bp within the 5′ UTR, transcripts for FL-L1b were clearly detected in CHOK1-M10 and CHOK1-N10 cells. Further analysis of four additional monochromosomal hybrid cell lines – two human/hamster hybrids CHOK1#8 and GM10926 (each containing an unrelated normal human chromosome 10) and two human/mouse hybrids GM11688 (containing a unrelated normal human chromosome 10) and ES-M10 (containing the mardel(10) chromosome) – showed positive transcription activities of FL-L1b in three of the hybrid lines (GM10926, GM11688, and ES-M10) but not in CHOK1#8 (Figure 3B; Table S2). No detectable transcriptional activity was detected for FL-L1a, FL-L1c, FL-L1d, FL-L1e and FL-L1f in any of these cell lines. These results indicated that the FL-L1b locus was actively transcribed both before and following neocentromere formation. In addition, it was of interest to note that FL-L1b was located within the central and largest CENP-A cluster (Figure 2B), and belonged to the active L1PA2 subfamily [20],[24],[25],[26].

To investigate whether the FL-L1b locus is the only active L1 element within the 10q25 neocentromeric chromatin, additional primers were specifically designed to target those truncated L1s that contained intact promoter sequences and also others that were greater than 4 kb in size identified within the 6-Mb genomic region (Figure 2A). These targets included five long truncated L1s with or without the promoter sequence (L1g–k) and other short orphan L1 promoter sequences (L1l–m). The results of RT-PCR analysis indicated no detectable transcripts from any of these L1 targets in the three monochromosomal hybrid cell lines assayed - CHOK1#8, CHOK1-N10, and CHOK1-M10 (Table S3).

Given that antisense transcription has been detected from the 5′ UTR of L1 elements [26],[27], we performed RT-PCR analysis on all promoter-containing FL-L1s and truncated L1s (L1a–f, i, k, l, m; Figure 2) within the 6-Mb region using primer sets each targeted to the 5′ upstream flanking sequence at one end and to the 5′ UTR of L1 at the other end. No antisense transcript could be detected for all promoter-containing L1s across the 6-Mb genomic region (Table S3). These results showed that, across the 6-Mb neocentromeric domain, active transcription was found only at the FL-L1b locus, and that the resulting RNA products were predominantly long sense transcripts of at least 415 bp in size.

### Incorporation of FL-L1b RNA Transcripts into the 10q25 Neocentromeric Chromatin

Next we investigated if the corresponding FL-L1b RNA transcripts were incorporated into the 10q25 neocentromeric chromatin. Chromatin immunoprecipitation was performed using a specific anti-CENP-A antibody. RNAs from both the input and immunoprecipitated fractions were isolated, reverse transcribed into cDNAs, and subjected to real-time quantitative PCR analysis using three independent primer sets targeted to the 5′ UTR of FL-L1b. A significant enrichment (*P*<0.001) of FL-L1b RNA in the CENP-A bound fractions was observed, as indicated by a 4 to 5 fold increase in the yield of PCR products (Figure 3D). In contrast, none of the negative control sequences, 18S, 5S, and β-actin, was enriched in the immunoprecipitated fractions. We have also performed similar RNA-ChIP experiments and analyzed the RNA-ChIP products using primers targeting to the other L1s (FL-L1a, -L1c, -L1d) as well as four genes (KIAA1600, TRUB1, GFRA1) that reside around the CENP-A-binding domain and detected no enrichment of any of these transcripts in the CENP-A chromatin of the CHOK1-M10 cells (Figure S2). Together, these results indicated the specific incorporation of the FL-L1b RNA into the CENP-A-associated chromatin of the 10q25 neocentromere.

### RNAi Knockdown of FL-L1b Transcripts Reduced the Mitotic Stability of Mardel(10) and the Level of CENP-A Protein at the 10q25 Neocentromere

**Figure 4:**
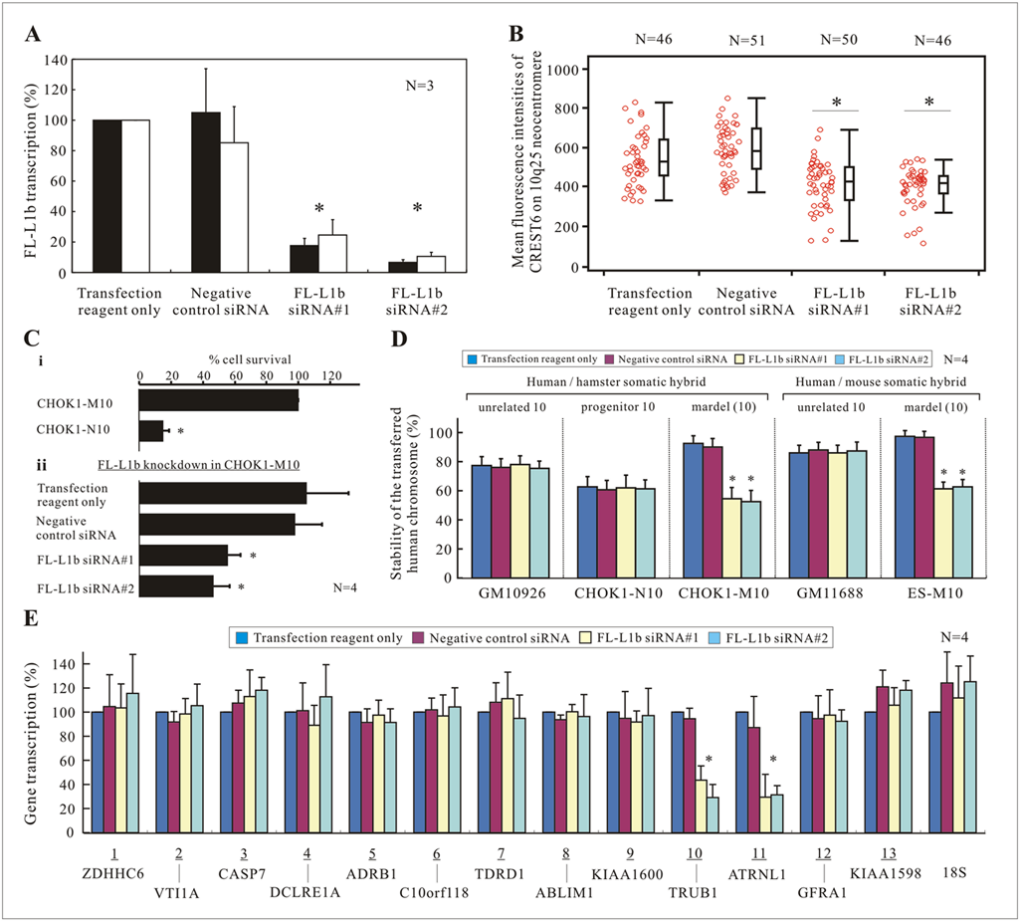
Transcriptional knockdown of FL-L1b and its associated effects on neocentromere structure and function. (A) Efficient RNAi knockdown of FL-L1b in CHOK1-M10. CHOK1-M10 cells were transfected with FL-L1b-specific siRNA oligonucleotide duplexes, FL-L1b siRNA#1 and siRNA#2, at a final concentration of 25 nM. Following 48 hours post transfection, FL-L1b RNA levels were assayed by quantitative RT-PCR analysis using primer sets FL-L1b.1 (black bars) and FL-L1b.3 (white bars). Both siRNA duplexes effectively knocked-down the transcription level of FL-L1b (*P*<0.05, indicated by the asterisks) by >80% (mean for n = 3, with SEM), compared to the transfection-reagent-only and Stealth siRNA low GC negative controls. (B) The structural integrity of 10q25 neocentromere in CHOK1-M10 cells after 24 hours post siRNA transfection was investigated by dual immunofluorescence/FISH analysis using an anti-CENP-A antiserum (CREST6) and a BAC probe (RP11-359H22) specific for the 10q25 neocentromeric region of mardel(10). After FL-L1b knockdown, the mean fluorescence intensity of CREST6 at 10q25 neocentromere was significantly reduced (by 20–30%; *P*<0.001, indicated by the asterisks). Each spot in the combined scatter/box plot represented the relative amount of CENP-A protein at 10q25 neocentromere in one metaphase spread, which was calculated by an average of 5 measurements normalized against the average signals on 15–20 CHO centromeres within each spread. (C) Zeocin kill-curve analysis. (i) Addition of 200 µg/ml Zeocin effectively killed CHOK1-N10 cells but did not affect the growth of CHOK1-M10 cells (n = 4, with SEM); CHOK1-M10 cells were resistant to Zeocin because the mardel(10) chromosome had been tagged with a Zeocin resistance gene. The majority of the CHOK1-N10 cells (>80%; *P*<0.05) were killed by 48 hours post Zeocin selection (n = 4, with SEM). % cell survival = total cell number under Zeocin selection/total cell number without Zeocin selection. (ii) A 40–50% reduction in cell viability (mean for n = 4, with SEM) was observed following knockdown of FL-L1b RNA and 48 hours of Zeocin selection. The differences in the % of cell survival in the L1b knockdown samples were statistically significant (*P*<0.05; indicated by the asterisks). (D) The stability of the neocentromeric mardel(10) chromosome or normal human chromosome 10 in the various hybrid cell lines was calculated as a percentage of the total number of cells containing a positive FISH signal of BAC RP11-359H22 after 48 hours of RNAi knockdown. FL-L1b transcription knockdown resulted in significantly reduced stability of the mardel(10) chromosome (mean for n = 4, with SEM) but not of the normal human chromosome 10 in both the hamster and mouse hybrid cell lines (*P*<0.01; indicated by the asterisks). (E) Quantitative RT-PCR analysis of 13 genes found within or surrounding the 10q25 neocentromeric chromatin (see Figure 2A) in CHOK1-M10 was carried out 24 hours post FL-L1b RNA knockdown. While most of the genes were unaffected, the transcriptional activities of 2 neighboring genes, TRUB1 and ATRNL1 (mean for n = 4, with SEM), were significantly reduced (by 60–70%; *P*<0.05, indicated by the asterisks).

To study the potential role of the FL-L1b RNA at the 10q25 neocentromere, we designed two sets of siRNA oligonucleotide duplexes (Figure S3) for the specific transcriptional knockdown of FL-L1b in the monochromosomal CHOK1-M10 hybrid line; the study of RNAi knockdown in a CHO background offered the advantage of minimizing any potential off-target RNAi knockdown effects because the CHO genome contained significantly diverged L1 elements. The transfection conditions for RNAi knockdown were optimized to achieve >80% reduction in the FL-L1b transcripts as compared to the transfection-reagent-only and Stealth siRNA negative controls (Figure 4A). Similar efficiency of FL-L1b transcriptional knockdown was also achieved in the other mouse/human and hamster/human somatic hybrids described above (data not shown).

To determine the cellular effects of the FL-L1b knockdown, a kill-curve analysis was performed on a CHOK1-M10 hybrid cell line containing a mardel(10) chromosome that had been tagged with a Zeocin resistance gene [13],[17]. At the optimal concentration of 200 µg/ml of Zeocin, the majority (>80%) of non-mardel(10)-containing CHOK1-N10 cells were killed 48 hours post Zeocin treatment, whereas the normal growth of CHOK1-M10 cells was not affected (Figure 4C-i). A significant loss of cell viability was observed in CHOK1-M10 following FL-L1b RNAi-knockdown, with the percentage of surviving CHOK1-M10 cells being reduced to approximately 50% compared to the transfection-reagent-only and Stealth siRNA negative controls 48 hours post Zeocin selection (Figure 4C). These results indicated a presumed FL-L1b-induced impairment of neocentromere function that has led to the loss of the Zeocin-resistant mardel(10) chromosome.

To further extend the Zeocin kill-curve results, a direct assessment of the loss of the mardel(10) chromosome following FL-L1b knockdown was determined by FISH (Fluorescence In Situ Hybridization) analysis using a mardel(10)-specific BAC probe. The stability of mardel(10) was greatly affected 48 hours post FL-L1b RNAi-knockdown, with a significant reduction from ∼95% to ∼55% in the CHOK1-M10 cell line, and from ∼100% to ∼60% in the mouse-human hybrid cell line ES-M10 (Figure 4D). Under similar conditions, the stability of the normal human chromosome 10 in control CHO-human (GM10926, CHOK1-N10) and mouse-human (GM11688) hybrid lines were not affected after FL-L1b transcriptional knockdown, suggesting that the loss of mardel(10) was directly linked to the effect of the FL-L1b knockdown on the neocentromere activity.

In order to further investigate the structural integrity of the neocentromere after FL-L1b transcriptional knockdown, a combined immunofluorescence and FISH analysis was performed on metaphase CHOK1-M10 cells using an anti-CENP-A antiserum (CREST6) and a BAC DNA probe (RP11-359H22) that hybridized to the 10q25 neocentromeric region of mardel(10). Cells were harvested at 24 hours following RNAi-knockdown in order to capture the early to intermediate stages of the disruption of neocentromere function prior to the complete loss of the mardel(10) chromosome. The mean fluorescence intensity of the CREST6 signals on the 10q25 neocentromere was reduced by 20 to 30% (*P*<0.001) after the FL-L1b transcriptional knockdown using either siRNA#1 or siRNA#2 (Figure 4B; examples of reduced CENP-A levels on 10q25 neocentromere post FL-L1b RNAi knockdown are shown in Figure S5). In some cases, the CREST signals on the 10q25 neocentromere were as low as 20% that of the control cells. In addition to the quantitative immunofluorescence data, ChIP and real-time PCR analysis was also performed using an anti-CENP-A antibody for analysis comparing the enrichments of CENP-A at the 10q25 neocentromere with and without FL-L1b RNAi knockdown in CHOK1-M10 cells (Figure S6). Consistently, the ChIP/PCR results showed a reduction of CENP-A protein at 10q25 neocentormere following RNAi knockdown of FL-L1b transcript, providing independent confirmation of the importance of FL-L1 transcript in regulating the structural integrity of 10q25 neocentromere.

### RNAi Knockdown of FL-L1b RNA Led to a Reduction in the Transcriptional Activities of Two Genes within and/or Neighboring the CENP-A-Associated Chromatin

We have previously reported that genes located across the 10q25 neocentromere region are transcriptionally competent [17]. Here, we used the transcription status of these genes as a measure to determine the effect of FL-L1b knockdown on the overall neocentromeric chromatin environment. The transcriptional levels of 13 actively transcribed genes within the 6-Mb 10q25 neocentromere region (see Figure 2) were determined by qRT-PCR analysis at 24 hours post FL-L1b RNAi-knockdown. While most of the genes were unaffected, the transcriptional activities of 2 genes, ATRNL1 (which spanned the CENP-A-binding domain) and TRUB1 (located outside the CENP-A domain, with its 5′-end CpG island being ∼410 kb away from the FL-L1b locus), were significantly reduced (by approximately 60–70%; *P*<0.05) after the FL-L1b transcriptional knockdown (Figure 4E).

To ensure that the FL-L1b RNAi knockdown-mediated mardel(10) chromosomal instability was not attributed to a reduction in the level of TRUB1 and/or ATRNL1 transcripts, siRNA oligonucleotide duplexes were designed to target these and two other immediately surrounding genes, KIAA1600 and GFRA1. Approximately 70–90% transcriptional knockdown was achieved for each of these genes in the CHOK1-M10 cells (Figure S4). No significant difference in the percentage cell survival was observed in the Zeocin kill-curve analysis, providing support for a specific role of FL-L1b rather than these genes in the maintenance of the mardel(10) stability (Figure S4).

## Discussion

### Enrichment of L1 Sequences at the 10q25 Neocentromere

Our earlier bioinformatic analysis revealed a >2.5-fold increase in the prevalence of L1 retrotransposons in the underlying DNA sequence of the 10q25 CENP-A-binding clusters [18]. In this study, we described the increased frequency of intact L1 segments and average length of L1 DNA within the 330-kb CENP-A domain. Across the 6-Mb region of the 10q25 neocentromeric chromatin, a concentrated cluster of four FL-L1s was found at the CENP-A-binding domain of the 10q25 neocentromere [18]. Furthermore, *in silico* analysis of other neocentromere sites (Figure S7) has revealed the presence of at least one FL-L1 element at the CENP-A-binding domain of five out of the six neocentromeres mapped to date [7],[8],[10]. The average FL-L1 density across these neocentromeres was also higher by 1.5 times compared to that of the human genome. These observations indicated a potential role of the L1 retrotransposon, particularly the full-length members (FL-L1s), in the regulation of neocentromeric chromatin.

### Active Transcription of FL-L1 Retrotransposon within the Core Neocentromere

In humans, active transcription and translation of L1 retrotransposons has been detected in a wide-range of cell types, including germ cells, tumours and transformed cell lines, and a smaller number of non-malignant somatic cells [21],[22],[28],[29],[30],[31],[32]. Importantly, multiple lines of evidence indicated that L1 RNAs are actively transcribed from full-length elements (∼6 kb in size) that contain an internal promoter, two ORFs, and a poly-A tail at the 3′ UTR [20],[21],[22],[23]. However, a detailed investigation of the transcriptional status of a single FL-L1 has not been described due to the technical difficulties associated with its repetitive nature. However, unlike tandemly-repeated satellite DNAs, which are highly homogeneous, L1 interspersed repeats are comparatively more diverged in sequence. Here, we took advantage of sequence divergence amongst the L1 repeats and designed oligonucleotide primers that targeted the diverged sites within a single FL-L1 retrotransposable element for RT-PCR and RNAi-knockdown analysis in monochromosomal somatic cell hybrids to determine its transcriptional activity and associated function – an undertaking that has not been previously described.

We determined the transcriptional status of all six FL-L1s and other non-full-length L1 targets within the 6-Mb genomic window spanning the core neocentromere. Interestingly, only one of them (i.e. FL-L1b) was actively transcribed from the mardel(10) in CHOK1-M10, although all six FL-L1s contained the internal promoter sequences (for sequence comparisons between transcriptionally active and silent FL-L1s assayed in this study, see Tables S4 and S5). Our previous study has described the active transcription of multiple genes within the broader 10q25 neocentromeric domain, including ATRNL1 that spanned the entire length of the CENP-A-associated chromatin [17]. However, it was uncertain if the core neocentromeric chromatin was permissive to active transcription given that the putative promoter of ATRNL1 was located outside the CENP-A domain. Here, based on the active transcription status of FL-L1b that is located within the central CENP-A-binding cluster at the 10q25 neocentromere, our study provided clear evidence for the permissibility of transcription within the core neocentromeric chromatin. More recently, this phenomenon of active transcription through the core centromere has also been demonstrated in α-satellite-containing human artificial chromosomes, where the CENP-A-associated domain was shown to spread into the adjacent transcriptionally active selectable marker gene [33],[34]. Furthermore, transcriptional competence of the core centromeric chromatin has also been described in *Oryza sativa* (rice) and *Zea mays* (maize) [35],[36]. These studies, including our current data, clearly show that CENP-A-associated chromatin is permissive to the transcription of genes and non-genic retrotransposable elements.

The pattern of FL-L1 transcription within the 6-Mb domain in the hamster-human hybrids CHOK1-N10 (containing the progenitor normal human chromosome 10) and GM10926 (containing an unrelated normal human chromosome 10) was identical to that found in CHOK1-M10. The formation of the 10q25 neocentromere did not significantly change the transcription level of FL-L1b, in consistent with our previous finding on the transcription competence of multiple genes located within this region [17]. Similar results were obtained from mouse-human hybrids GM11688 (containing an unrelated normal human chromosome 10) and ES-M10 (containing the neocentromeric mardel(10) chromosome), indicating that the active transcription of FL-L1b was not affected by differences in species background. Interestingly, FL-L1b transcription was not detected in one of the normal human chromosome 10 in the CHOK1#8 cell line – an observation that may be explained by differential epigenetic silencing or by mutations at the promoter or upstream regulatory sequences of the CHOK1#8 FL-L1b DNA.

### FL-L1 RNA Is a Structural and Functional Epigenetic Component of the Core Neocentromeric Chromatin

Using RNA-ChIP-qPCR analysis, we showed that FL-L1b single-stranded RNA transcripts were incorporated as part of the ribonucleoprotein component of the CENP-A-associated domain. Interestingly, the presence of long single-stranded centromeric RNA transcripts including *CentC* satellite repeats and *CRM* retrotransposons in *Zea mays* [36], *160B/Athila2* retrotransposon in *Arabidopsis thaliana* [37], *PRAT* satellite repeats in *Palorus ratzeburgi* [38], and α-satellite repeats in humans [39] were also reported in recent studies. Furthermore, chromatin immunoprecipitation experiments in *Zea mays* and humans independently showed that these centromeric RNA transcripts were associated with the core centromeric chromatin [36],[39]. Together, these results indicated that a pool of single-stranded RNA could be directly transcribed from the satellite repeats (and centromere-specific retrotransposons) of the normal centromeres or the L1 retrotransposon of a neocentromere and subsequently incorporated into the core centromeric/neocentromeric chromatin.

The functional role of FL-L1b RNA at the 10q25 neocentromere was determined by RNAi knockdown of FL-L1b in human/mouse and human/hamster monochromosomal hybrid lines. FISH and/or Zeocin kill-curve analysis indicated that FL-L1b knockdown led to a significant reduction (by ∼40–50%) of the mitotic stability of mardel(10) and the compromised structural integrity of the 10q25 neocentromere. These FL-L1b knockdown-mediated mitotic effects at the 10q25 neocentromere were fast and similar to the rapid response previously described in RNAi knockdown or conditional knockout of core centromere proteins, such as CENP-A [40], CENP-H [41],[42],[43] and CENP-K [44]. Our results therefore demonstrate a functional significance of L1 RNA transcripts at the core neocentromere region which has not been fully defined in previous studies.

In addition to the two FL-L1b siRNA duplexes, we have included the analysis of siRNA duplexes that targeted four genes spanning and surrounding the CENP-A-associated region. None of these siRNAs exerted any effect on mardel(10) stability, as indicated by the cell viability assay (Figure S4). More specifically, RNAi knockdown of ATRNL1, a gene that spanned across the CENP-A-associated domain, did not result in any compromise in the functional integrity of the 10q5 neocentromere. These data indicate that the FL-L1b RNAi-induced mardel(10) instability is likely to be a result of the depletion of FL-L1b RNA transcripts rather than due to indirect effects arising from the recruitment of chromatin remodelling or modifying complexes to the 10q25 neocentromere via the RNAi pathway.

The precise functional role(s) of FL-L1 RNA transcripts at the core neocentromeric chromatin remains to be delineated. Transcription at the FL-L1 locus and/or the L1 transcript itself may act as an early-specification epigenetic signal for the recruitment of CENP-A nucleosomes. Interestingly, the transcriptional knockdown of FL-L1b leads to a more ‘closed’ local chromatin state, as indicated by the reduction in the transcription of two surrounding genes ATRNL1 and TRUB1. At the 10q25 neocentromere, the transcription activity may facilitate the process of histone replacement by partially disassembling the nucleosomes to provide a more ‘open’ chromatin structure [45] for subsequent deposition of CENP-A nucleosomes. The recent identification of GATA-type transcription factor Ams2, which promotes the centromere localization of CENP-A in *Schizosaccharomyces pombe*, also provides supports toward a role of transcription in defining a centromere state [46].

Rather than the transcriptional activity itself, it is also possible that the FL-L1b RNA transcript may serve as a specific epigenetic signal at the 10q25 neocentromere since by RNAi knockdown several neighbouring genes did not affect the mitotic stability of mardel(10). Although this hypothesis remains to be tested, the underlying process may be similar to the function of long *Xist* RNA in promoting the establishment of a specialized chromatin state such as the incorporation of macroH2A during X-inactivation [47],[48]. Alternatively, the chromatin-bound FL-L1b RNA at the 10q25 neocentromere may be involved in the formation of a flexible ribonucleoprotein complex that brings together and/or stabilizes the proteins of the core neocentromere, as suggested by the observed CENP-A delocalization after FL-L1b RNAi knockdown. Nonetheless, it is interesting to note that the FL-L1b locus is being actively transcribed from both the progenitor chromosome 10 and the neocentric mardel(10). The absence of active CENP-A recruitment to the FL-L1b locus on the progenitor chromosome suggests that the FL-L1b transcript is unlikely the sole epigenetic specification determinant for CENP-A recruitment. The transcribed FL-L1b locus and/or FL-L1b RNA-bound chromatin may require additional players (e.g. specific RNA-binding proteins) in recruiting CENP-A for the formation of a neocentromere.

The reduction in the transcriptional activity of the two genes surrounding CENP-A domain (*ie.* ATRNL1 and TRUB1) following FL-L1b knockdown indicated that the FL-L1b RNA could be regulating a larger genomic domain than that of the CENP-A-associated chromatin. It is unknown how these FL-L1 RNA transcripts mediate such long-range chromosomal effects, however, it is interesting that this extended genomic domain overlaps with a region of high L1 DNA content (using the human genome average as the baseline threshold) (Figure 2B). The incorporation of FL-L1b RNA into the neocentromeric chromatin may potentially involve a simple base pair recognition mechanism [49], similar to what has been described for the assembly of the telomerase complex by telomerase RNA or the formation of heterochromatin structure by short interfering siRNA [50],[51]. In future studies, the identification of potential chromatin remodelling proteins that interact with the centromeric or neocentromeric RNA transcripts should shed new light on the epigenetic mechanisms of regulation of centromere/neocentromere architecture and function.

Increasing evidence now point to neocentromere formation as the underlying mechanism for centromere repositioning that underpins karyotype evolution and speciation [6],[52]. The elucidation of the molecular mechanisms of neocentromere formation will not only provide important insights into the inherent epigenetic determinants that initiate *de novo* centromere assembly, but will also provide a better understanding of the operating mechanisms for centromere repositioning and karyotype evolution.

## Materials and Methods

### Cell Cultures

The somatic hybrid cell lines were cultured as previously described [17],[53]. These include (i) human/hamster monochromosomal hybrid CHOK1#8, CHOK1-N10 and CHOK1-M10, containing unrelated chromosome 10, progenitor chromosome 10, and mardel(10) respectively; (ii) human/mouse mardel(10)-containing monochromosomal hybrid ES-M10 [14],[17]. Two additional somatic hybrid cell lines, GM10926 (CHOK1 background) and GM11688 (mouse A9 background), each containing an unrelated human chromosome 10, were obtained from the Human Genetic Cell Repository of Coriell Institute of Medical Research and were cultured in Ham's Kao and Michayluk medium (KAO) supplemented with 10% dialyzed FCS (Gibco BRL) at 37°C and Ham's F12 Medium/DMEM (1∶1 mixture) 2 mM L-glutamine, 10% FCS with 500 µg/ml Geneticin (Gibco) at 37°C. 200 µg/ml Zeocin (Invitrogen) was added into the media for selection of the mardel(10) chromosome in CHOK1-M10 and ES-M10.

### Zeocin Kill-Curve Cell Viability Assay

A time-course experiment was first performed to determine the time duration required to kill the non-resistant CHOK1 cells (CHOK1-N10) at 200 µg/ml of Zeocin. The transcription-knockdowns of FL-L1b and other genes of interest were performed by siRNA transfection of the relevant siRNA oligonucleotide duplexes for 48 hours at 25 nM in CHOK1-M10. Subsequently, cells were incubated with 200 µg/ml of Zeocin for an additional 48 hours following RNAi knockdown. The number of viable cells was determined by staining with Trypan Blue (0.8 mM Trypan Blue in 1× PBS) for 5 minutes at room temperature and counting with a hemocytometer under the microscope. The mitotic stability of the mardel(10) was calculated as the ratio of the percentage of viable cells under Zeocin selection to the number of viable cells without Zeocin selection.

### *In Silico* Sequence Analysis

The genomic location of each chromatin domain and the sequence characteristics were determined using the UCSC Genome Browser (http://genome.ucsc.edu.au) May 2004 builds and its in-build RepeatMasker track [54]. Full-length L1s were identified and annotated using the online L1Base software package (http://l1base.molgen.mpg.de/) [55]. Specifically, several key features were analyzed and these included (1) general characteristics, such as the GC content, target site duplications, intactness scores, the polyadenylation signal, and the presence of poly-A tails; (2) classifications of L1s; (3) 5′UTR promoter features and the conservations of transcription factor binding sites; (4) the conservation of amino acid residues in the two ORFs (Table S4).

In the L1Base program, the ‘intactness score’ was calculated for the query FL-L1 sequence. One point was awarded to every conserved sequence feature (according to the consensus L1 sequence) that was known to affect the transcriptional and/or translational activity [55]. The transcriptionally active FL-L1b had an intactness score of 25, being the highest of the six FL-L1s (Table S5A). As for the 100 bp internal promoter [56],[57] within the 5′ UTR, the nucleotide sequence conservation of the six FL-L1s (FL-L1a–g) ranges from 72.3 to 91.6% and FL-L1b ranked the equal highest of the six FL-L1s (Table S5B). FL-L1b also contained all of the known conserved transcription factor binding sites within the 5′ UTR, while more than one mutation was found within the 5′ UTR of the other FL-L1s. In addition, a CpG island that was potentially important for transcriptional regulation was present within FL-L1b. Other noted sequence features of FL-L1b that could contribute to its transcription and functional activities were listed following: (1) FL-L1b contained an intact polyadenylation signal and a relatively long poly-A tail, which were important for mRNA maturation and subsequent protein translation; (2) FL-L1b was the only FL-L1 of the 6 FL-L1s with no ORF frame shifts or mutations at the important amino acid residues analysed; (3) FL-L1b belonged to the retrotranspositionally competent Ta subfamily and was flanked by 15 bp target-site duplications (Table S5 C to F).

### RNA-ChIP

RNA chromatin immunoprecipitation was performed as described in [58] with slight modifications. RIPA Buffer (50 mM Tris-Cl, pH 7.5, 1% NP-40, 0.5% sodium deoxycholate, 0.05% SDS, 1 mM EDTA, 150 mM NaCl, 1 tablet of Roche Complete Protease Inhibitor per 10 ml of RIPA buffer) was used for cell lysis and immunoprecipitation was performed using a rabbit polyclonal anti-mouse CENP-A antibody at 1∶500 dilution [15]. Immunocomplex recovery was achieved following two washes with RIPA High Stringency Wash Buffer (50 mM Tris-HCl pH 7.5, 1% Nonidet P-40, 1% sodium deoxycholate, 0.1% SDS, 1 mM EDTA, 0.1 mM PMSF, 1 tablet of Roche Complete™ Protease Inhibitor per 10 ml buffer) containing 250 mM and 500 mM NaCl in stepwise manner. Elution of RNA was performed with RNA-ChIP Elution Buffer (50 mM Tris-HCl pH 7.5, 5 mM EDTA, 1% SDS, 10 mM dithiothreitol) and reverse cross-linked at 70°C for 45 minutes. Total RNA was then isolated and subsequently subjected to quantitative PCR analysis.

### Quantitative RT-PCR

Total RNA was extracted using either the RNeasy Mini Kit (Qiagen) for transcription detection assays or TRIZOL reagent (Invitrogen) for RNA-ChIP. TURBO DNA-free Kit (Ambion) was used to remove possible contaminating DNA. cDNA synthesis was performed using Transcriptor First Strand cDNA Synthesis Kit (Roche). Quantitative RT-PCR was carried out using SYBR Green PCR Master Mix (Applied Biosystems) on 7300 or 7900HT Real-Time PCR System (Applied Biosystems) according to manufacturer's instructions. cDNA equivalent to 10 ng RNA was amplified with 150 nM forward and reverse primers in a 25 µL reaction (for primer sequences, see Table S6). Dissociation curves were performed to confirm specific amplifications without primer dimer formation. Samples were also subjected to gel electrophoresis analysis to confirm that the PCR products were of expected size. For the transcription assay of the FL-L1s, sequencing experiments were also performed to confirm the identity of each RT-PCR product. For calculations and statistics in the analysis, see below.

The comparative C_T_ method was used for data analysis in transcription detection assay and quantitative ChIP-PCR analysis. The ΔC_T_ value was calculated as [ΔC_T_ = C_T_ (test gene/genomic segment)−C_T_ (control gene/genomic segment)]. The C_T_ value of each test gene/segment was normalized against the C_T_ value of control gene/segment, either 5S (for DNA-ChIP-qPCR analysis) or β-actin (for transcription assay and RNA-ChIP-qPCR analysis) to give the ΔC_T_ value. The ΔΔC_T_ value was calculated as [ΔΔC_T_ = ΔC_T(test cell line)_−ΔC_T(control cell line)_] for transcription analysis, or [ΔΔC_T_ = ΔC_T(before siRNA knockdown)_−ΔC_T(after siRNA knockdown)_] for transcription knockdown assay, and [ΔΔC_T_ = ΔC_T(input)_−ΔC_T(bound)_] for ChIP-qPCR analysis, respectively. Relative fold-difference in transcription activity was expressed as 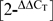 in transcription analysis and transcription knockdown assays. Relative-binding value in ChIP-qPCR analysis was calculated by 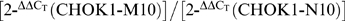.

### Transcriptional Knockdown by siRNA Transfection

Two sets of Stealth siRNA oligonucleotide duplexes targeting FL-L1b were designed using the online BLOCK-iT RNAi Designer software (Invitrogen). In contrast, siRNA oligonucleotide duplexes targeting genes KIAA1600, TRUB1, ATRNL1, and GFRA1 were obtained as pre-designed Stealth Select siRNA (Invitrogen). Sequences of the siRNA oligonucleotide duplexes are listed in Table S6. CHOK1-M10 cells were seeded in 6-well culture plates without antibiotic selection at a density of 2×10^4^ cells/well, 24 hours prior to siRNA transfection. Transcriptional knockdown was performed by transfecting cells with Stealth siRNA oligonucleotide duplexes (Invitrogen) at a final concentration of 25 nM in DMEM (Dulbecco's Modified Eagle's Medium) using 2.5 ng/µl Lipofectamine 2000 (Invitrogen) for a period of 24 to 48 hours according to the manufacturer's instructions. The effects of RNAi knockdown of FL-L1b and other target genes were assayed by quantitative RT-PCR. Stealth siRNA Negative Control Low GC Duplex (Invitrogen) was also included as control for sequence independent RNAi knockdown effects.

### Combined Immunofluorescence/FISH and Quantification of Immunofluorescence Signals

Immunofluorescence [59] and FISH [13] were performed as previously described. Anti-centromere autoimmune serum CREST6 (which predominantly recognize CENP-A protein) and RP11-359H22 BAC were used for the identification of 10q25 neocentromere on mardel(10) [13]. Metaphase spreads were visualized using an Imager M1 microscope (Zeiss) and the digital images were captured by the AxioCam MRm camera (Zeiss). CREST6 immunofluorescence signals on 10q25 neocentromere were quantified and normalized against CHO centromeres in CHOK1-M10 cells using AxioVision software version V4.6.1.0 (Zeiss).

The quantification of CREST6 immunofluorescence signals was performed following FL-L1b RNAi knockdown. A circular area of defined size (diameter of 2 µm) was selected around the centromere of interest. Total intensity (I) of each pixel within the delineated area was determined and defined as arbitrary fluorescence unit (a.f.u.). Digital images obtained from immunofluorescence analysis were nonsaturating and auto-corrected for background removal. Non-specific background signal (I^BK^) for each metaphase spread was calculated by the average arm intensity from five chromosomes and subsequently subtracted from the total intensity (I). Average signal intensity of 15–20 endogenous CHO centromeres (I^CHO^) from each spread was calculated and used as normalization control to correct for the variation in hybridization between spreads. The ratio of CREST6 fluorescence intensities (R) on 10q25 neocentromere to CHO centromeres was calculated using the following equation: R = (I^M10^−I^BK^)/(I^CHO^−I^BK^). The mean fluorescence intensity of CREST6 (M) on 10q25 neocentromere was calculated using the following equation: M = R×I^CHOALL^. I^CHOALL^ represents the average intensity for all CHO centromeres (∼750) calculated for each treatment in the RNAi knockdown experiments.

## Supporting Information

Figure S1 DNA-ChIP-qPCR analysis. DNA-ChIP was performed using a specific anti-CENP-A antibody as previously described [18]. 250 ng of input or immunoprecipitated DNA was subjected to quantitative PCR analysis using three independent primer sets L1.ORF1, L1.ORF2a, and L1.ORF2b (targeting to the L1 consensus sequence L1.2). L1 genomic sequences were significantly enriched in the CENP-A-bound fraction (*P*<0.05) in CHOK1-M10 when compared to CHOK1-N10, ranging from approximately 4 to 6 fold increase in relative binding (mean for n = 4, with SEM). In contrast, none of negative control sequences, 18S, 5S, and hamster HC2sat repeat was enriched in the pull-down fractions, indicating a specific enrichment of L1 sequences in the CENP-A chromatin.
(2.2 MB TIF)

Figure S2 RNA-ChIP-qPCR analysis. RNA-ChIP analysis was performed using a specific anti-CENP-A antibody followed by quantitative RT-PCR analysis. Positive control FL-L1b (amplified using L1b.1 primer set) and negative controls 18S, 5S and ACTB were included in the experiment. Test genomic targets included (A) four transcribed genes (KIAA1600, TRUB1, ATRNL1 and GFRA1) and (B) three other FL-L1s (FL-L1a, -L1c, -L1d) within or surrounding the CENP-A-binding domain. The PCR amplification of the active FL-L1b retrotransposon and the four test genes occurred at much earlier cycles (with Ct values ranging between 28–30 cycles) than the three silent FL-L1s (Ct values ranging between 36–40 cycles). Relative binding values (mean for n = 3, with SEM) on the Y-axis represent the fold-enrichment of the target sequence in CHOK1-M10 compared to that of CHOK1-N10. Except for FL-L1b, none of the other loci tested showed RNA enrichment at the CENP-A-binding domain.
(3.0 MB TIF)

Figure S3 Design of siRNA sequences for RNAi knockdown of FL-L1b. (A) Using the online Invitrogen RNAi BLOCK-iT algorithm (https://rnaidesigner.invitrogen.com/), two sets of oligonucleotide duplexes, FL-L1b siRNA#1 and siRNA#2, were successfully designed each targeting to a specific site within the ORF2 region of FL-L1b. (B) Output from *in silico* BLAT (BLAST-like alignment tool, USCS Genome Browser http://genome.ucsc.edu/) analysis showed that each of these siRNA duplexes had only one hit of 100% homology to the human chromosome 10 and no additional homologous sequences could be found in the other mammalian genomes analysed, including *Mus musculus* (mouse), *Rattus norvegicus* (rat), and *Gallus Gallus* (chicken).
(0.9 MB TIF)

Figure S4 Transcriptional knockdown of four different genes within or surrounding the CENP-A-binding domain. (A) Approximately 70–90% transcription knockdown (mean for n = 3, with SEM) was achieved for (i) KIAA1600 (ii) TRUB1 (iii) ATRNL1 (iv) GFRA1 genes by the corresponding siRNAs at a final concentration of 25 nM in CHOK1-M10 cells after 48 hours (*P*<0.05, indicated by the asterisks). (B) No significant difference in % cell survival (mean for n = 4, with SEM) was observed 48 hours post Zeocin selection following gene knockdown compared to the transfection-reagent-only and Stealth siRNA negative controls.
(4.3 MB TIF)

Figure S5 Transcriptional knockdown of FL-L1b and its effect on neocentromere structure and function. CHOK1-M10 cells were transfected with either (A) control siRNA oligonucleotide duplexes or (B) FL-L1b-specific siRNA oligonucleotide duplex FL-L1b-siRNA#2 (B), at a final concentration of 25 nM. Following 24 hours post transfection, the structural integrity of 10q25 neocentromere in CHOK1-M10 cells was investigated first by immunofluorescence analysis using an anti-CENP-A antiserum (CREST6; A–B i–ii) followed by FISH analysis using a BAC probe (RP11-359H22; A–B iii) specific for the 10q25 neocentromeric region of mardel(10) (as indicated by the arrow). The non-specific background signal intensity (IBK) was calculated as the CREST signal intensity on the chromosome arms (from the average of five chromosomes). The CREST6 signal intensities on both CHO endogenous centromeres (ICHO; from the average of 10 centromeres) and 10q25 neocentromere on mardel(10) (IM10) were determined. The ratio of CREST6 fluorescence intensities (R) on 10q25 neocentromere to CHO centromeres was calculated using the following equation: R = (IM10-IBK)/(ICHO-IBK). After FL-L1b knockdown, the fluorescence intensity of CREST6 at 10q25 neocentromere was reduced by 49.3% [calculated as (R _L1b knockdown_−R _control_.)/R _control_×100%].
(4.1 MB TIF)

Figure S6Transcription knockdown of FL-L1b followed by RNA-ChIP-qPCR analysis. RNAi knockdown of FL-L1b was performed by transfecting CHOK1-M10 cells with either control siRNA oligonucleotide duplexes or FL-L1b-specific siRNA oligonucleotide duplexes, (A) FL-L1b-siRNA#1 and (B) siRNA#2, at a final concentration of 25 nM. Following 24 hours post transfection, ChIP was performed using a specific anti-CENP-A antibody. Subsequent quantitative real-time PCR analysis was carried out using three independent primer sets A3.1, A3.2 and A4 each targeting to a genomic fragment of approximately 200 bp to the previously described CENP-A-binding clusters A3 or A4 (third and fourth clusters counting from the left as shown in Figure 2B) within the 330-kb CENP-A-binding domain of the 10q25 neocentromere [18]. Four independent experiments were shown and on average (mean for n = 4, with SEM), the relative binding of the three neocentromeric CENP-A-associated genomic fragments in the L1b-knockdown cells was reduced by approximately 50% (siRNA#1) or 75% (siRNA#2), when compared to the transfection-reagent-only control. This provides added support to the immunofluorescence/FISH data shown in Figure S5 for a reduction in the binding of CENP-A proteins at the 10q25 neocentromere following FL-L1b knockdown. The comparative C_T_ method was used for data analysis. The ΔC_T_ value was calculated as [ΔC_T_ = C_T_ (test segment)−C_T_ (control segment)]. The C_T_ value of each test segment (A3.1, A3.2, and A4) was normalized against the C_T_ value of control segment (either C1 or C2) to give the ΔC_T_ value. The ΔΔC_T_ value was calculated as [ΔΔC_T_ = ΔC_T(input)_−ΔC_T(bound)_]. The fold-enrichment in CENP-A binding was expressed as 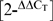. The relative changes in CENP-A binding levels were calculated by 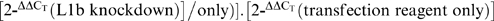.(4.3 MB TIF)

Figure S7 Distribution of FL-L1s within and surrounding the CENP-A-binding domain of six different neocentromeres. To date, the CENP-A-binding domain, ranging in size from 131 to 464 kb (red boxes), has been mapped for six different neocentromeres using ChIP-array analysis [19]. FL-1s (green bars) were identified within a 2-Mb genomic segment surrounding each of the CENP-A domains. According to the L1Base database, 11798 FL-L1s are present in the human genome, giving an average density of 0.381 FL-L1 per 100 kb. Here, we performed bioinformatic analysis on the previously published six CENP-A domains and found that the average FL-L1 density for these regions was 0.572 per 100 kb, which is 1.5 higher than that of the human genome. Interestingly, at least one full-length L1 was present within the CENP-A-binding domain of five out of the six neocentromeres.
(2.3 MB TIF)

Table S1 Sequence characteristics of L1s across a 6-Mb genomic region of the 10q25 neocentromere.
(0.02 MB XLS)

Table S2 Transcription status of FL-L1s within a 6-Mb genomic region of the 10q25 neocentromere.
(0.02 MB XLS)

Table S3 Sequence characteristics and transcription status of L1s within a 6-Mb genomic region of the 10q25 neocentromere.
(0.02 MB XLS)

Table S4 Sequence features of FL-L1s analysed using the online L1Base program.
(0.02 MB XLS)

Table S5 Sequence characteristics of FL-L1s across the 10q25 neocentromere.
(0.04 MB XLS)

Table S6 Oligonucleotide primers and siRNA duplexes.
(0.03 MB XLS)
