# Correction: LINE Retrotransposon RNA Is an Essential Structural and Functional Epigenetic Component of a Core Neocentromeric Chromatin

**DOI:** 10.1371/annotation/3b497aec-b7d3-442e-9086-751251f649dd

**Published:** 2009-02-12

**Authors:** Anderly C. Chueh, Emma L. Northrop, Kate H. Brettingham-Moore, K. H. Andy Choo, Lee H. Wong

There were errors in the Author Contributions. The correct contributions are: Conceived and designed the experiments: ACC KHAC LHW. Performed the experiments: ACC ELN LHW. Analyzed the data: ACC ELN KHBM KHAC LHW. Contributed reagents/materials/analysis tools: KHAC LHW. Wrote the paper: ACC KHAC LHW. Technical advice and discussion: ACC ELN KHBM KHAC LHW.

