# Supplementary figures and images for: LINE Retrotransposon RNA Is an Essential Structural and Functional Epigenetic Component of a Core Neocentromeric Chromatin
Source: PLoS Genet. 2009 Jan 30;5(1):e1000354. doi: 10.1371/journal.pgen.1000354 (PMC2625447; doi:10.1371/journal.pgen.1000354)

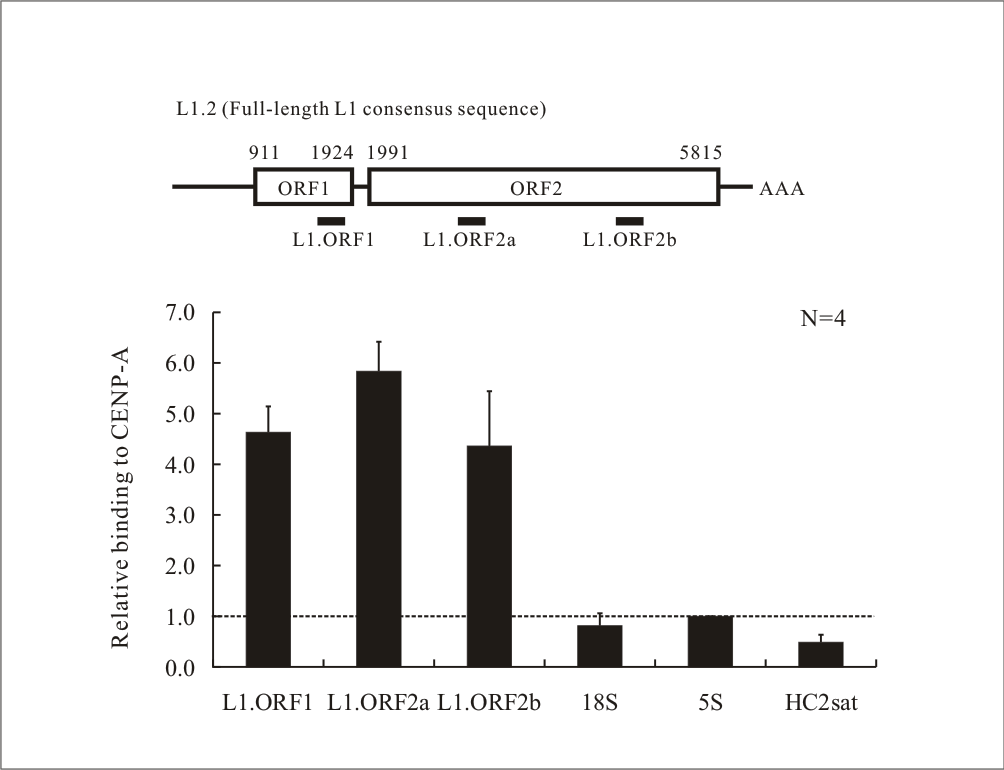

Supplement: Figure S1 — DNA-ChIP-qPCR analysis. DNA-ChIP was performed using a specific anti-CENP-A antibody as previously described [18]. 250 ng of input or immunoprecipitated DNA was subjected to quantitative PCR analysis using three independent primer sets L1.ORF1, L1.ORF2a, and L1.ORF2b (targeting to the L1 consensus sequence L1.2). L1 genomic sequences were significantly enriched in the CENP-A-bound fraction (P<0.05) in CHOK1-M10 when compared to CHOK1-N10, ranging from approximately 4 to 6 fold increase in relative binding (mean for n = 4, with SEM). In contrast, none of negative control sequences, 18S, 5S, and hamster HC2sat repeat was enriched in the pull-down fractions, indicating a specific enrichment of L1 sequences in the CENP-A chromatin. (2.2 MB TIF) [file pgen.1000354.s001.tif]

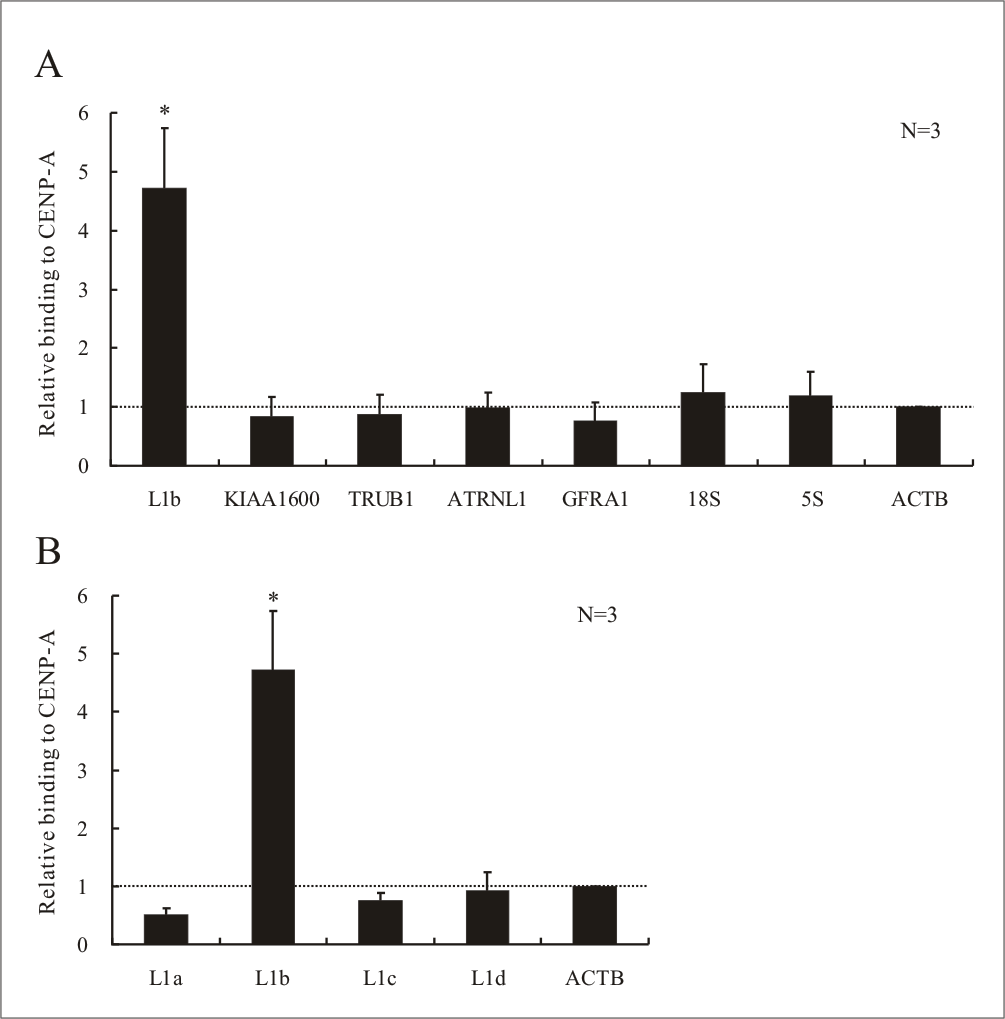

Supplement: Figure S2 — RNA-ChIP-qPCR analysis. RNA-ChIP analysis was performed using a specific anti-CENP-A antibody followed by quantitative RT-PCR analysis. Positive control FL-L1b (amplified using L1b.1 primer set) and negative controls 18S, 5S and ACTB were included in the experiment. Test genomic targets included (A) four transcribed genes (KIAA1600, TRUB1, ATRNL1 and GFRA1) and (B) three other FL-L1s (FL-L1a, -L1c, -L1d) within or surrounding the CENP-A-binding domain. The PCR amplification of the active FL-L1b retrotransposon and the four test genes occurred at much earlier cycles (with Ct values ranging between 28–30 cycles) than the three silent FL-L1s (Ct values ranging between 36–40 cycles). Relative binding values (mean for n = 3, with SEM) on the Y-axis represent the fold-enrichment of the target sequence in CHOK1-M10 compared to that of CHOK1-N10. Except for FL-L1b, none of the other loci tested showed RNA enrichment at the CENP-A-binding domain. (3.0 MB TIF) [file pgen.1000354.s002.tif]

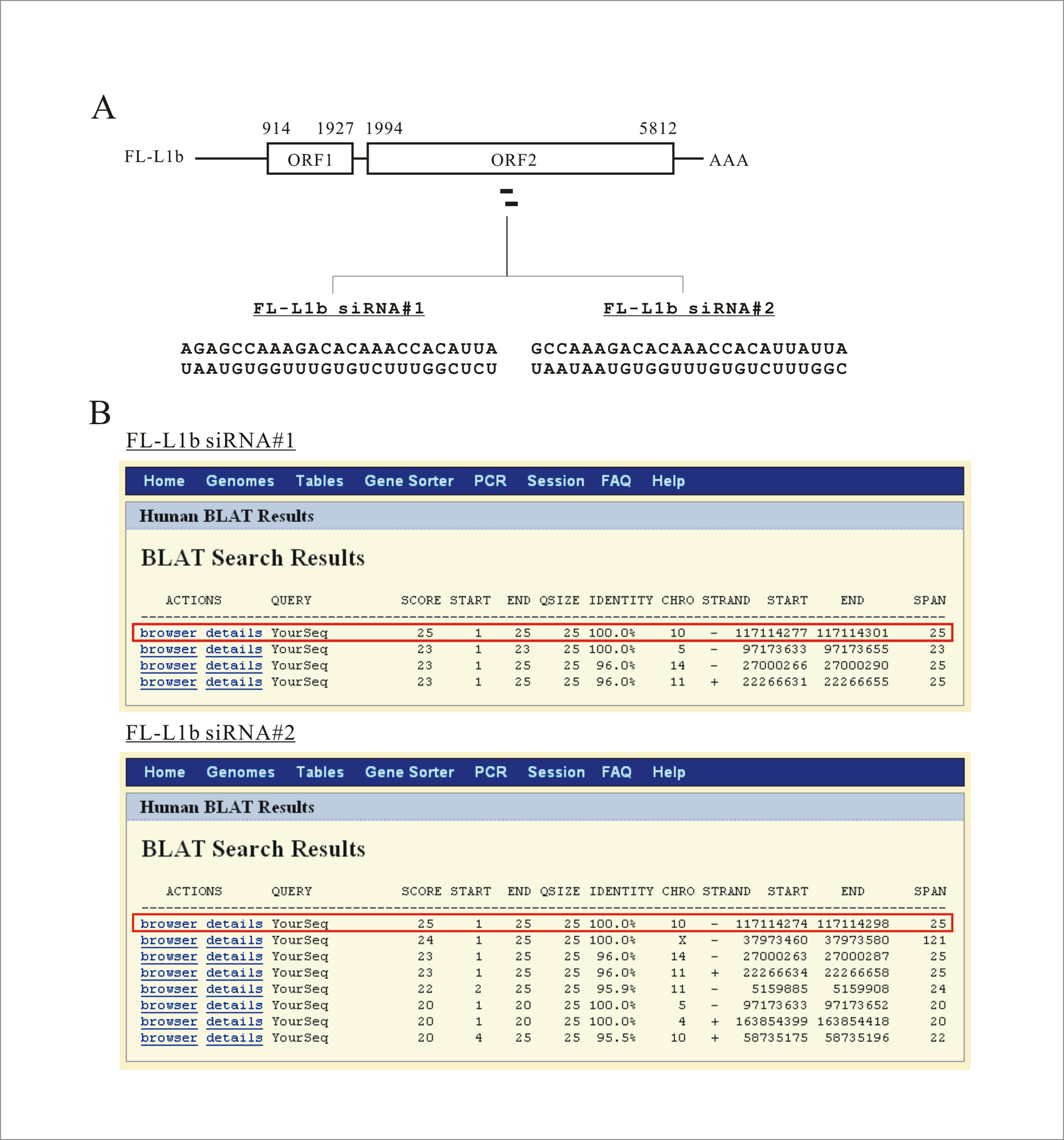

Supplement: Figure S3 — Design of siRNA sequences for RNAi knockdown of FL-L1b. (A) Using the online Invitrogen RNAi BLOCK-iT algorithm (https://rnaidesigner.invitrogen.com/), two sets of oligonucleotide duplexes, FL-L1b siRNA#1 and siRNA#2, were successfully designed each targeting to a specific site within the ORF2 region of FL-L1b. (B) Output from in silico BLAT (BLAST-like alignment tool, USCS Genome Browser http://genome.ucsc.edu/) analysis showed that each of these siRNA duplexes had only one hit of 100% homology to the human chromosome 10 and no additional homologous sequences could be found in the other mammalian genomes analysed, including Mus musculus (mouse), Rattus norvegicus (rat), and Gallus Gallus (chicken). (0.9 MB TIF) [file pgen.1000354.s003.tif]

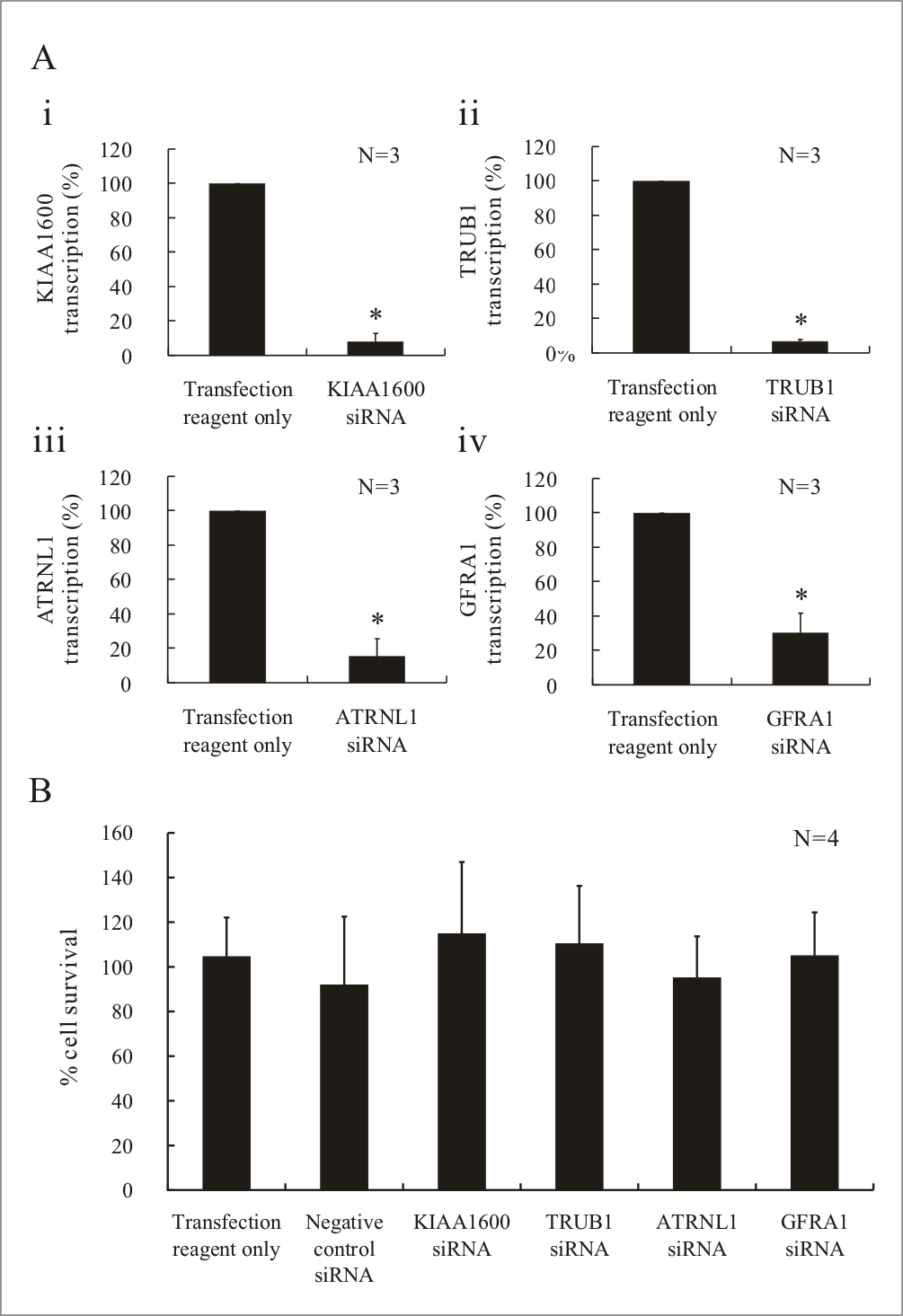

Supplement: Figure S4 — Transcriptional knockdown of four different genes within or surrounding the CENP-A-binding domain. (A) Approximately 70–90% transcription knockdown (mean for n = 3, with SEM) was achieved for (i) KIAA1600 (ii) TRUB1 (iii) ATRNL1 (iv) GFRA1 genes by the corresponding siRNAs at a final concentration of 25 nM in CHOK1-M10 cells after 48 hours (P<0.05, indicated by the asterisks). (B) No significant difference in % cell survival (mean for n = 4, with SEM) was observed 48 hours post Zeocin selection following gene knockdown compared to the transfection-reagent-only and Stealth siRNA negative controls. (4.3 MB TIF) [file pgen.1000354.s004.tif]

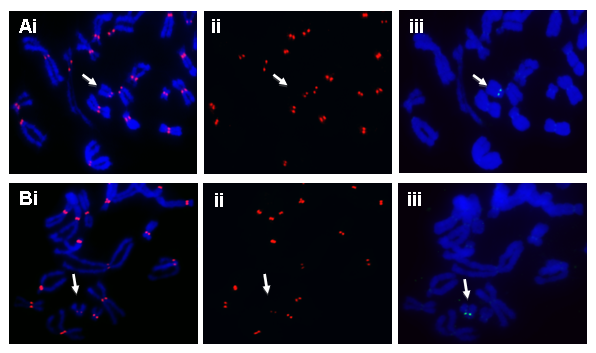

Supplement: Figure S5 — Transcriptional knockdown of FL-L1b and its effect on neocentromere structure and function. CHOK1-M10 cells were transfected with either (A) control siRNA oligonucleotide duplexes or (B) FL-L1b-specific siRNA oligonucleotide duplex FL-L1b-siRNA#2 (B), at a final concentration of 25 nM. Following 24 hours post transfection, the structural integrity of 10q25 neocentromere in CHOK1-M10 cells was investigated first by immunofluorescence analysis using an anti-CENP-A antiserum (CREST6; A–B i–ii) followed by FISH analysis using a BAC probe (RP11-359H22; A–B iii) specific for the 10q25 neocentromeric region of mardel(10) (as indicated by the arrow). The non-specific background signal intensity (IBK) was calculated as the CREST signal intensity on the chromosome arms (from the average of five chromosomes). The CREST6 signal intensities on both CHO endogenous centromeres (ICHO; from the average of 10 centromeres) and 10q25 neocentromere on mardel(10) (IM10) were determined. The ratio of CREST6 fluorescence intensities (R) on 10q25 neocentromere to CHO centromeres was calculated using the following equation: R = (IM10-IBK)/(ICHO-IBK). After FL-L1b knockdown, the fluorescence intensity of CREST6 at 10q25 neocentromere was reduced by 49.3% [calculated as (R L1b knockdown−R control.)/R control×100%]. (4.1 MB TIF) [file pgen.1000354.s005.tif]

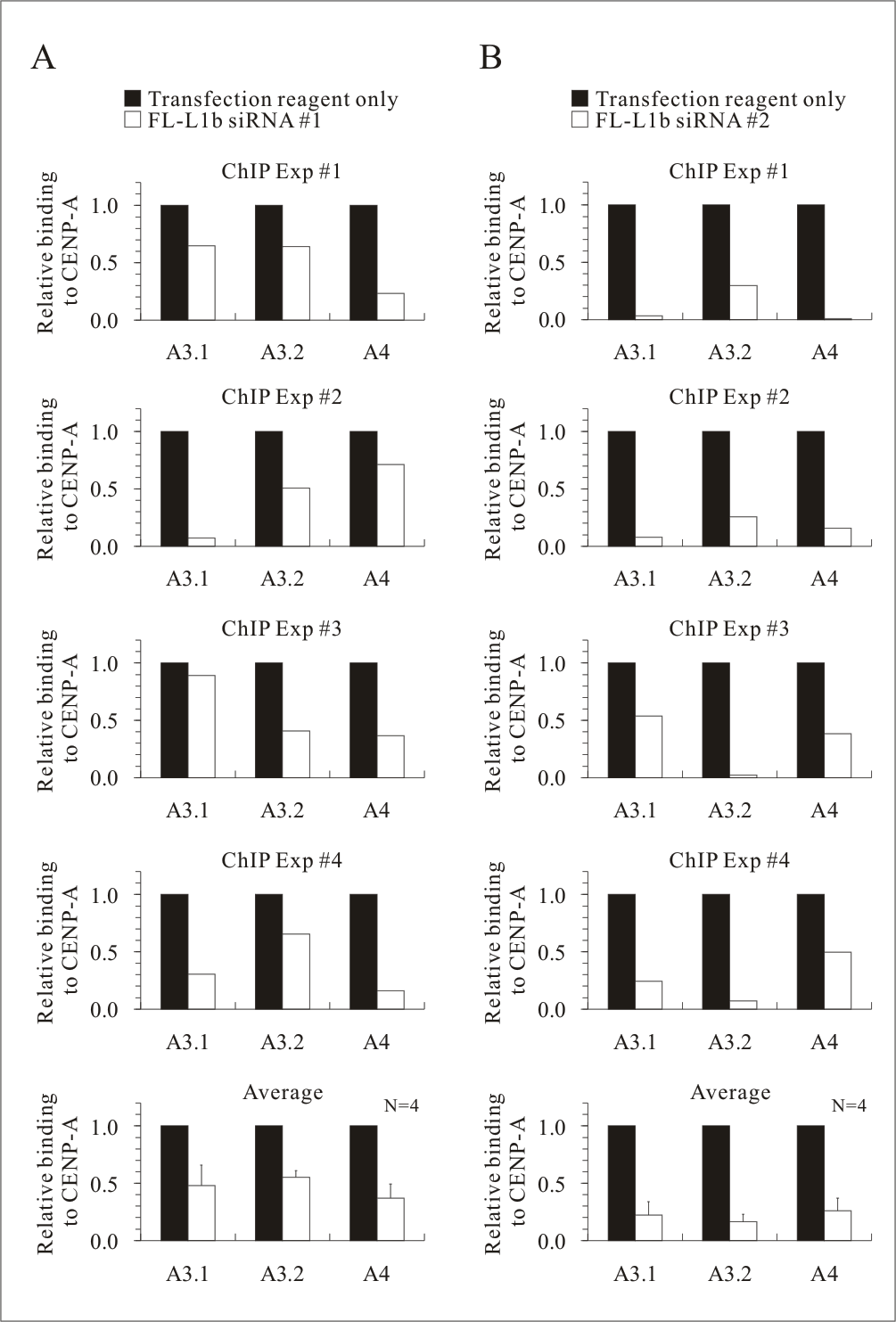

Supplement: Figure S6 — Transcription knockdown of FL-L1b followed by RNA-ChIP-qPCR analysis. RNAi knockdown of FL-L1b was performed by transfecting CHOK1-M10 cells with either control siRNA oligonucleotide duplexes or FL-L1b-specific siRNA oligonucleotide duplexes, (A) FL-L1b-siRNA#1 and (B) siRNA#2, at a final concentration of 25 nM. Following 24 hours post transfection, ChIP was performed using a specific anti-CENP-A antibody. Subsequent quantitative real-time PCR analysis was carried out using three independent primer sets A3.1, A3.2 and A4 each targeting to a genomic fragment of approximately 200 bp to the previously described CENP-A-binding clusters A3 or A4 (third and fourth clusters counting from the left as shown in Figure 2B) within the 330-kb CENP-A-binding domain of the 10q25 neocentromere [18]. Four independent experiments were shown and on average (mean for n = 4, with SEM), the relative binding of the three neocentromeric CENP-A-associated genomic fragments in the L1b-knockdown cells was reduced by approximately 50% (siRNA#1) or 75% (siRNA#2), when compared to the transfection-reagent-only control. This provides added support to the immunofluorescence/FISH data shown in Figure S5 for a reduction in the binding of CENP-A proteins at the 10q25 neocentromere following FL-L1b knockdown. The comparative CT method was used for data analysis. The ΔCT value was calculated as [ΔCT = CT (test segment)−CT (control segment)]. The CT value of each test segment (A3.1, A3.2, and A4) was normalized against the CT value of control segment (either C1 or C2) to give the ΔCT value. The ΔΔCT value was calculated as [ΔΔCT = ΔCT(input)−ΔCT(bound)]. The fold-enrichment in CENP-A binding was expressed as . The relative changes in CENP-A binding levels were calculated by . (4.3 MB TIF) [file pgen.1000354.s006.tif]

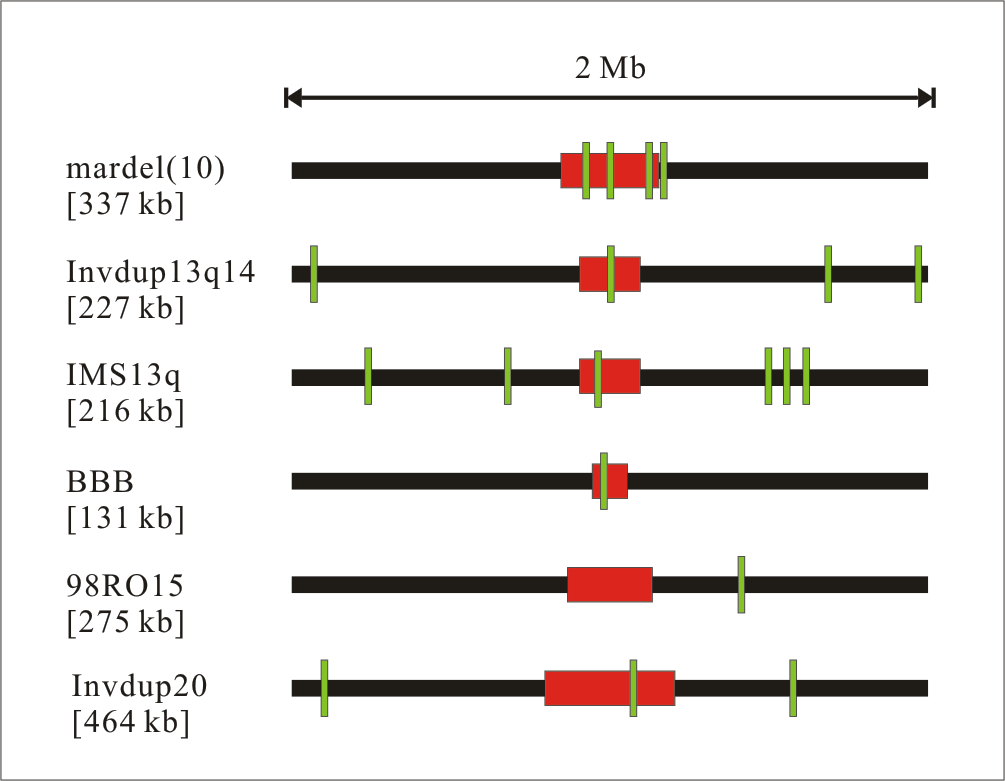

Supplement: Figure S7 — Distribution of FL-L1s within and surrounding the CENP-A-binding domain of six different neocentromeres. To date, the CENP-A-binding domain, ranging in size from 131 to 464 kb (red boxes), has been mapped for six different neocentromeres using ChIP-array analysis [19]. FL-1s (green bars) were identified within a 2-Mb genomic segment surrounding each of the CENP-A domains. According to the L1Base database, 11798 FL-L1s are present in the human genome, giving an average density of 0.381 FL-L1 per 100 kb. Here, we performed bioinformatic analysis on the previously published six CENP-A domains and found that the average FL-L1 density for these regions was 0.572 per 100 kb, which is 1.5 higher than that of the human genome. Interestingly, at least one full-length L1 was present within the CENP-A-binding domain of five out of the six neocentromeres. (2.3 MB TIF) [file pgen.1000354.s007.tif]
